# Optimizing Urgency of Information through Resource Constrained Joint Sensing and Transmission

**DOI:** 10.3390/e24111624

**Published:** 2022-11-09

**Authors:** Zhuoxuan Ju, Parisa Rafiee, Omur Ozel

**Affiliations:** Department of Electrical and Computer Engineering, George Washington University, Washington, DC 20052, USA

**Keywords:** urgency of information, information freshness, resource constraints, Lyapunov optimization

## Abstract

Applications requiring services from modern wireless networks, such as those involving remote control and supervision, call for maintaining the timeliness of information flows. Current research and development efforts for 5G, Internet of things, and artificial intelligence technologies will benefit from new notions of timeliness in designing novel sensing, computing, and transmission strategies. The age of information (AoI) metric and a recent related urgency of information (UoI) metric enable promising frameworks in this direction. In this paper, we consider UoI optimization in an interactive point-to-point system when the updating terminal is resource constrained to send updates and receive/sense the feedback of the status information at the receiver. We first propose a new system model that involves Gaussian distributed time increments at the receiving end to design interactive transmission and feedback sensing functions and develop a new notion of UoI suitable for this system. We then formulate the UoI optimization with a new objective function involving a weighted combination of urgency levels at the transmitting and receiving ends. By using a Lyapunov optimization framework, we obtain a decision strategy under energy resource constraints at both transmission and receiving/sensing and show that it can get arbitrarily close to the optimal solution. We numerically study performance comparisons and observe significant improvements with respect to benchmarks.

## 1. Introduction

As demand from wireless networks exponentially increases to enable emerging technologies, the timeliness of data delivery and adaptation to the context of information becomes essential for improved quality of service and experience in time-sensitive applications. To this end, the measurement and improvement of the timeliness of data delivery and the effective adaptation to the context of delivered data have been fundamental challenges that researchers and practitioners have worked on actively in recent years. The age of information (AoI) is a well-known metric to measure the timeliness of data from the perspective of the nodes receiving or consuming data [1] and is expressed as the time elapsed since the generation of the latest received data. Although AoI has received much interest as a metric representing the freshness of information, new metrics are needed to address nonlinearity in the aging of data and time-varying value or context associated with flowing data. As a matter of fact, context-based applications (e.g., automatic driving and artificial intelligence) and nonlinear age [2,3,4] (as in many IoT applications) require a departure from AoI definition and analysis. Toward this end, the references [5,6] recently proposed an urgency of information (UoI) framework by combining the timeliness and context associated with information updates. In these papers, UoI was formally defined as the product of context-aware weight and the cost resulting from real-time estimation error in a Gaussian dynamical system, the latter being a well-known nonlinear function of AoI. UoI expression can be expressed in mathematical form as follows:

(1) $$\begin{array}{c} F\left(t\right)=w_{t}\delta \left(C\left(t\right)\right), \end{array}$$

where $w_{t}$ is the nonnegative coefficient representing the context or value at a specific time *t*, δ(.) is the cost function, and C(t) is the instantaneous cost measuring the urgency. This formulation subsumes the typical definition of AoI. If C(t) increases by one each time an update is not received, then the common AoI problem can be formulated as $w_{t}=1$ and δ(Q(t))=U(t)C(t), where U(t) is an indicator that shows whether the information is updated or not. In our current paper, we will pursue a similar metric whereby the urgency level is represented by a coefficient $w_{t}$, which will be set as an independent, identically distributed random process that shows how crucial the status information is at a specific moment *t*. In addition, we will pursue a quadratic cost function. This formulation enables us to analyze error increments and connect the proposed framework to the classical AoI problem.

The UoI framework in this paper will be designed to measure the expected performance degradation as a weighted sum of expected staleness or informativeness of the latest sensed Gaussian process at the receiving end with respect to the transmitter and the lack of synchrony between them, maintained by status updating from the transmitting end to the receiving end. Our goal is to build a systematic understanding on the interaction of feedback sensing and update transmission to maintain improved UoI levels measuring the synchrony and informativeness of information at one side about the other side when both actions are resource constrained. We will employ Lyapunov optimization tools to address this crucial problem.

Lyapunov optimization methods and tools have been well-known to various research communities to control queues and more generally dynamical systems in a near-optimal sense. In the context of queuing theory, the state of a system at a particular time is the vector of realizations of error variables which can easily be brought in queue forms by lower bounding it by zero and studied for upper bounding the optimal cost. Typically, the cost function is defined to take smaller value when the system moves toward the desirable states. System stability is achieved by taking control actions that make the Lyapunov drift in the negative direction toward zero. The key requirement is that all the queues and virtual queues in the system are mean rate stable [7,8]. In addition, the target function is achieved by taking control actions that minimize the Lyapunov penalty. However, because of the system stability awareness, the solution always has a gap with the optimal solution. Due to its general applicability in queuing theory, Lyapunov optimization is also used in AoI analysis and optimization. Ref. [9] used Lyapunov optimization to identify the tradeoff between AoI, accuracy, and completeness with the constrained throughput optimization problem. Ref. [10] used Lyapunov optimization to jointly minimize the average cost of sampling and transmitting status updates by users over a wireless channel subject to average AoI constraints.

Our work’s motivation is rooted in AoI research that was presented in the recent past. We next aim to cover some of the literature that relates to the proposed research in this paper. The references [11,12] address varying source update frequency and [13,14] address service rate in various queuing models. In the wireless network scenario, the scheduling algorithms for optimizing AoI is studied extensively, such as those considering the channel state [15,16], throughput [17,18,19], energy harvesting [20,21,22], and average resource constraints [23,24], multiple sources [25,26,27,28], and multiple channels [29,30,31,32] to name a few. Ref. [33] studied the calculation and iterative process of AoI in combination with queuing theory and gave the analytic formula of average AoI under the random scheduling strategy. Ref. [34] explored the impact of service rate on average AoI under fixed deadline constraints and random exponential deadline constraints. Regarding link scheduling in wireless networks, ref. [35] studied the link-scheduling problem for every time slot under periodic data updates, and proposed random, greedy, Lyapunov optimization, Whittle Index, and other strategies for link scheduling to optimize the average AoI of the network. Ref. [36] proposed offline and online scheduling algorithms based on the Markov decision process for the random data arrival scenario.

Feedback is also an essential factor in wireless communication scenarios and can influence the AoI performance significantly. In particular, it is well known that the feedback may help maintain expedient processing, non-repetitive transmission, and hence, energy efficiency in wireless transmission. For the case of battery-based non-energy harvesting devices, it is also vital to schedule appropriate transmission and sensing strategies to prolong the device’s life. As a result, the role of feedback and energy cost in AoI analysis and optimization has received much interest from the research community (see e.g., [37,38,39,40,41]). Additionally, ref. [42] proves that the AoI and energy-harvesting scheduling strongly differ with or without the feedback. Refs. [43,44] minimized the AoI when the sensor uses ON/OFF schemes with energy harvesting nodes. Ref. [45] focused on the extreme cases of one unit battery and infinite battery situations to minimize the average peak AoI with energy constraints. Most recently, the paper [46] provided an analysis of feedback cost in AoI optimization over a point-to-point channel and determined specific conditions when feedback may or may not be useful for AoI optimization.

Decisions to sense/receive updates under energy constraints have also been of interest to AoI researchers. In particular, energy constraints can limit the chance of sensing new data and hence cause AoI to increase. In this context, refs. [47,48] proposed the joint scheduling of sense and transmission schemes to optimize the average peak AoI in an energy-harvesting system. In this paper, we will combine the concept of feedback and sensing, which means that the system will decide whether to sense the feedback information as input. As other related research, refs. [49,50] studied the value of information (VoI) in status update systems, and compared the performance of VoI with AoI. We also refer the reader to the related paper [51]. Based on the idea that AoI is only important when the receiver performs a query, refs. [52,53] proposed the age of information at query (QAoI) and optimized the QAoI.

In this paper, we will extend the UoI optimization framework in [6] to an interactive scenario by considering sensing/receiving costs at the updating terminal under energy resource constraint by using a Lyapunov optimization framework. Resource constraints in receiving/sensing the feedback can be interpreted as a limitation due to processing or energy to make it available for decision making on update transmission. Our motivation can be compared to that of [46] as well, which assumes the cost of feedback is incurred at the receiving end. This new problem calls for coordinated decisions to sense the feedback from the receiver and transmit the update to the receiver. Additionally, we need to account for relativity with respect to the transmitter and receiver sides and measure urgency by using a weight representing their importance under resource constraints. Our framework will address these new issues.

As the main contributions of this paper, we extend the UoI optimization framework by using a new definition that addresses the interactive nature of the setting when transmitting and receiving/sensing information is costly and average resource constraints are present on both actions. Constructing the objective function by assigning different weights to the urgency levels at the transmitting and receiving terminals, we determine jointly optimal scheduling of transmission and receiving/sensing the feedback by using a Lyapunov optimization framework. We obtain the Lyapunov gap and show that the result can be made arbitrarily close to the optimal solution. Our simulation results show that the proposed algorithm performs significantly better than two benchmark schemes, namely the greedy and AoI optimal algorithms.

The rest of the paper is organized as follows. In Section 2, we present the system model of the UoI problem. In Section 3, we formulate the UoI problem and analyze it. In Section 4, we offer numerical results to show the behavior of the solution. Finally, we conclude this paper in Section 5 by summarizing our contributions and discussing future directions.

## 2. System Model

**Figure 1:**
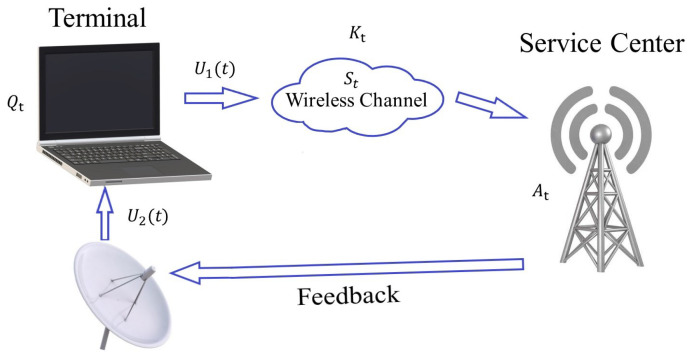
Systemmodel with joint transmission and feedback reception.

We consider the system model in Figure 1. Here, the time is slotted: t=1,2,…,T. The information-carrying signal in the service center and terminal, $A_{t}$ and $Q_{t}$, are as follows:

(2) $$\begin{array}{c} A_{t+1}=\left(1-U_{2}\left(t\right)\right)A_{t}+K_{t} \end{array}$$

(3) $$\begin{array}{c} Q_{t+1}=\left(1-S_{t}U_{1}\left(t\right)\right)Q_{t}+U_{2}\left(t\right)A_{t}. \end{array}$$

The variable $K_{t}∼N\left(0,\sigma^{2}\right)$ represents the increments added to the information-carrying signal $A_{t}$ and is a Gaussian random variable independent over time and other variables. For convenience, we take the variable $A_{1},Q_{1}∼N\left(0,\sigma^{2}\right)$; however, the initial conditions are assumed given and do not determine the outcome as long as they come from a well-behaving distribution that makes the expectations well defined (c.f. Lemma 3 below). $U_{1}\left(t\right),U_{2}\left(t\right)\in \left\{0,1\right\}$ are decision variables to determine whether to transmit an update and sense the feedback, respectively. Equation (2) represents the evolution of information at the receiver with respect to the sensing at the transmitter. When $U_{2}\left(t\right)=1$, the sensing action is activated and the information at both ends are synchronized except an additive Gaussian noise due to causality and one time slot difference. The Equation (3) represents the evolution of the information at the transmitter with respect to the receiver side. These two equations represent the interaction between the transmitter and the receiver. Note that if the transmission or sensing does not happen, i.e., if $U_{1}\left(t\right)S_{t}=0$ or $U_{2}\left(t\right)=0$, then $Q_{t}$ or $A_{t}$, respectively, will become noisier. This is at the heart of the urgency of information notion we pursue in this paper. When a transmission does not happen (due to not transmitting or a channel erasure), the synchrony between the two sides, represented by $Q_{t}$, is not affected as long as a new sensing action is not taken. At the beginning of the *t*th time slot, the terminal first decides $U_{1}\left(t\right)\in \left\{0,1\right\}$ to determine whether to transmit the information-carrying variable $Q_{t}$ to the service center or not. The transmission takes one time slot and goes through an erasure-type wireless channel represented by $S_{t}$ with a fixed failure transmission rate *p*. In particular, $S_{t}=1$ if the transmission is successful and $S_{t}=0$ otherwise. At the same time, the service center feeds back $A_{t}$ to the terminal, which also takes one time slot with no failure rate. At the end of the *t*th time slot, the feedback arrives at the terminal, and the terminal will decide $U_{2}\left(t\right)\in \left\{0,1\right\}$ to determine whether to sense the feedback or not. We can, in principle, let $A_{t}$ and $Q_{t}$ evolve as $\max\{\left(1-U_{2}\left(t\right)\right)A_{t}+K_{t},0\}$ and $\max\{\left(1-S_{t}U_{1}\left(t\right)\right)Q_{t}+U_{2}\left(t\right)A_{t},0\}$ with nonnegative initial values. These versions bring these system states to the form of queues with potentially dependent arrivals and departures. Our Lyapunov drift plus penalty-based analysis will be applicable for both versions. We therefore prefer to keep them as in (2) and (3) in the ensuing analysis.

Now we can elicit our optimization problem $P_{1}$ to minimize an upper bound of average UoI:

(4) $$\begin{array}{cc} \; & \min_{\pi_{t}}\lim_{T\to \infty }sup\frac{1}{T}\sum_{t=0}^{T-1}E\left[w_{t}({Q_{t}}^{2}+M{A_{t}}^{2})\right] \end{array}$$

(5) $$\begin{array}{cc} s.t\; & \lim_{T\to \infty }sup\frac{1}{T}\sum_{t=0}^{T-1}E\left[U_{1}\left(t\right)\right]\leq \varphi_{1} \end{array}$$

(6) $$\begin{array}{cc} \; & \lim_{T\to \infty }sup\frac{1}{T}\sum_{t=0}^{T-1}E\left[U_{2}\left(t\right)\right]\leq \varphi_{2}, \end{array}$$

where $\pi_{t}$ is the set of sequence of decisions $\pi_{t}=\left\{U_{1}\left(t\right),U_{2}\left(t\right)\right\}$, $w_{t}$ is the nonnegative weight of urgency modeled as an i.i.d. random variable, *M* is the weight of the relative error of the variable $A_{t}$ at the transmitter side, $\varphi_{1}$ is the energy (or frequency) constraint on transmission, and $\varphi_{2}$ is the energy (or frequency) constraint on sensing. In order to satisfy the average transmission/sensing frequency constraints (5) and (6), we define the virtual queues $H_{t}$ and $G_{t}$ as follows, which are both initialized at 0:

(7) $$\begin{array}{c} H_{t+1}=\max\left\{H_{t}-\varphi_{1}+U_{1}\left(t\right),\;0\right\} \end{array}$$

(8) $$\begin{array}{c} G_{t+1}=\max\left\{G_{t}-\varphi_{2}+U_{2}\left(t\right),\;0\right\}. \end{array}$$

Next, let us consider the evolution of the transmission virtual queue: If the terminal decides to transmit at time slot *t*, the transmission virtual queue $H_{t}$ will increase by $1-\varphi_{1}$. Otherwise, it will decrease by $\varphi_{1}$. As a result, the longer the virtual queue, the more transfers will be performed. The virtual queue of sensing $G_{t}$ evolves similarly. Therefore, these two virtual queues can appropriately express the usage of the historical transmission/sensing frequency.

## 3. Optimizing the Urgency of Information in $P_{1}$

In this section, we will systematically develop a Lyapunov optimization framework for optimizing an upper bound for the solution of $P_{1}$. We summarize the notations we use throughout the rest of the paper in Table 1.

### 3.1. Lyapunov Function Definitions

In order to use the Lyapunov optimization framework, we will first define the Lyapunov drift function $\Delta_{t}$ by using the quadratic sum of system states:

(9) $$\begin{array}{c} L_{t}=\frac{1}{2}VH_{t}^{2}+\frac{1}{2}ZG_{t}^{2}+\frac{1}{2}{\theta Q_{t}}^{2}+\frac{1}{2}{\beta A_{t}}^{2}, \end{array}$$

where V,Z,θ and β are the weights for different variables, which represent different importance levels of the stability of the queues or system states $H_{t},G_{t},Q_{t}$ and $A_{t}$, respectively. In our analysis, we use the terms “queue” or “system state” interchangeably. Although the evolution of $A_{t}$ and $Q_{t}$ in (2) and (3) can take negative values, we can redefine them by lower bounding their evolution by zero and make their definitions suitable as a queue with arrivals and departures potentially depending on the control actions. However, none of the analysis steps we take in this paper will be affected by this redefinition, as the Lyapunov analysis we present essentially optimizes a bound on the system performance. We therefore continue using the original definitions (2) and (3). The Lyapunov drift function for this system can be expressed as

(10) $$\begin{array}{c} \Delta_{t}=E\left[L_{t+1}-L_{t}|Q_{t},n_{t},H_{t},G_{t},w_{t+1}\right], \end{array}$$

where $n_{t}$ is the number of time slots since the last time we decide to sense the feedback. It is obvious that in *t*th time slot, the terminal has a knowledge of $H_{t},G_{t},Q_{t}$. However, the terminal cannot access the specific value of $A_{t}$ because the latest estimation error arrived at the service center at the end of (t−1)st time slot. Nevertheless, the terminal is aware of the number of time slot since the last time it decides to sense $n_{t}$, which can be expressed as:

(11) $$\begin{array}{c} n_{t+1}=\left(1-U_{2}\left(t\right)\right)n_{t}+1. \end{array}$$

As a result, the terminal will decide whether to sense based on the number of time slot since the last time it decided to sense $n_{t}$ rather than the error in the service center $A_{t}$.

**Lemma** **1.** *In each time slot t, given the error in terminal $Q_{t}$, urgency weight at the next time slot $w_{t+1}$, the number of time slots since the last time terminal decides to sense $n_{t}$, virtual queue length $H_{t}$ and $G_{t}$, set $Y_{t}=\left\{Q_{t},n_{t},H_{t},G_{t},w_{t+1}\right\}$, we can obtain an upper bound on the Lyapunov drift $\Delta_{t}$ as*

(12) $$\begin{array}{cc} \Delta_{t}\leq & \frac{1}{2}\left(V+Z\right)+\frac{1}{2}\beta \sigma^{2}-V\varphi_{1}H_{t}-Z\varphi_{2}G_{t}+\left(VH_{t}-\frac{1}{2}\theta p{Q_{t}}^{2}\right)E\left[U_{1}\left(t\right)|Y_{t}\right]\\ \; & +\left(ZG_{t}+\frac{1}{2}\theta n_{t}\sigma^{2}-\frac{1}{2}\beta n_{t}\sigma^{2}\right)E\left[U_{2}\left(t\right)|Y_{t}\right]. \end{array}$$

**Proof.**  See Appendix A.    □

Denote the penalty in the *t*th time slot by $f_{t}$. Because of causality, $U_{1}\left(t\right),U_{2}\left(t\right)$ will affect UoI in (t+1)st time slot. Therefore, we let $f_{t}=Rw_{t+1}\left({Q_{t+1}}^{2}+M{A_{t+1}}^{2}\right)$, where *R* is the weight of the UoI compared with system stability and the remaining terms represent UoI at t+1.

**Lemma** **2.** *If we set the penalty in the tth time slot as $f_{t}=Rw_{t+1}({Q_{t+1}}^{2}+M{A_{t+1}}^{2})$, and the average of the weight of the urgency as $\tilde{w}$. The Lyapunov drift plus penalty function is upper bounded as:*

(13) $$\begin{array}{cc} \Delta_{t}+E\left[f_{t}|Y_{t}\right]\leq & \frac{1}{2}\left(V+Z\right)+\frac{1}{2}\beta \sigma^{2}+R\tilde{w}\left(Q_{t}^{2}+M\sigma^{2}+Mn_{t}\sigma^{2}\right)-V\varphi_{1}H_{t}-Z\varphi_{2}G_{t}\\ \; & +\left(VH_{t}-\frac{1}{2}\theta p{Q_{t}}^{2}-R\tilde{w}pQ_{t}^{2}\right)E\left[U_{1}\left(t\right)|Y_{t}\right]\\ \; & +\left(ZG_{t}+\frac{1}{2}\theta n_{t}\sigma^{2}-\frac{1}{2}\beta n_{t}\sigma^{2}+\left(1-M\right)R\tilde{w}n_{t}\sigma^{2}\right)E\left[U_{2}\left(t\right)|Y_{t}\right]. \end{array}$$

**Proof.**  See Appendix B.    □

**Lemma** **3.** *If $E\left[L_{0}\right]<\infty$, and $\Delta_{t}+E\left[f_{t}\right]\leq C$, where C is a constant, then all the queues and virtual queues in the system are mean rate stable.*

**Proof.**  See Appendix C.    □

### 3.2. Finding Appropriate Weights for the System

Next, we are going to find the optimal value of the weight parameters θ and β to minimize the right hand side of (13) to the extent possible. Note that it is feasible to use a stationary randomized scheme that independently transmits and senses with probability $\varphi_{1}$ and $\varphi_{2}$ at each time slot, which translates to $E\left[U_{1}\left(t\right)\right]=\varphi_{1}$ and $E\left[U_{2}\left(t\right)\right]=\varphi_{2}$. As a result, we reorder (13) to get

(14) $$\begin{array}{cc} E\left[L_{t+1}-L_{t}+f_{t}|Y_{t}\right]\leq & (R\tilde{w}M+\frac{1}{2}\beta {)\sigma }^{2}+\frac{1}{2}\left(V+Z\right)+\left(-\frac{1}{2}\theta p\varphi_{1}-R\tilde{w}{p\varphi }_{1}+R\tilde{w}\right){Q_{t}}^{2}\\ \; & +\left(\frac{1}{2}\left(\theta -\beta \right)n_{t}\sigma^{2}+R\tilde{w}M+\left(1-M\right)R\tilde{w}\varphi_{2}\right){n_{t}\sigma }^{2}. \end{array}$$

To make the right hand side of (14) no larger than a constant, we want the coefficients of ${Q_{t}}^{2}$ and ${n_{t}\sigma }^{2}$ no larger than 0. For the coefficient of ${Q_{t}}^{2}$,

(15) $$\begin{array}{cc} -\frac{1}{2}\theta p\varphi_{1} & -R\tilde{w}{p\varphi }_{1}+R\tilde{w}\leq 0\\ \theta & \geq \frac{2}{{p\varphi }_{1}}\left(1-{p\varphi }_{1}\right)R\tilde{w}. \end{array}$$

For the coefficient of ${n_{t}\sigma }^{2}$,

(16) $$\begin{array}{cc} \frac{1}{2}\left(\theta -\beta \right)n_{t}\sigma^{2}+ & R\tilde{w}M+\left(1-M\right)R\tilde{w}\varphi_{2}\leq 0\\ \beta & \geq \theta +2\left(\frac{1}{\varphi_{2}}-1\right)R\tilde{w}M+2R\tilde{w}. \end{array}$$

As a result, we take the value of the parameters θ and β as

(17) $$\begin{array}{c} \theta =\frac{2}{{p\varphi }_{1}}\left(1-{p\varphi }_{1}\right)R\tilde{w} \end{array}$$

(18) $$\begin{array}{c} \beta =\frac{2}{{p\varphi }_{1}}R\tilde{w}+2\left(\frac{1}{\varphi_{2}}-1\right)R\tilde{w}M. \end{array}$$

Put the value of the parameters θ and β back to (14), then we can get the upper bound of $E\left[L_{t+1}-L_{t}+f_{t}|Y_{t}\right]$ as

(19) $$\begin{array}{c} E\left[L_{t+1}-L_{t}+f_{t}|Y_{t}\right]\leq \left(\frac{1}{{p\varphi }_{1}}+\frac{M}{\varphi_{2}}\right)R\tilde{w}\sigma^{2}+\frac{1}{2}\left(V+Z\right). \end{array}$$

Note that the right hand of (19) is a constant, which means that all the queues and virtual queues in the system are mean rate stable under above derived conditions.

### 3.3. Deriving Lyapunov Optimal Decisions

We now minimize the upper bound in the RHS of (13), which is actually in the following form:

(20) $$\begin{array}{cc} \min_{\pi_{t}} & \;\;\left(VH_{t}-\frac{1}{2}\theta p{Q_{t}}^{2}-Rw_{t+1}pQ_{t}^{2}\right)U_{1}\left(t\right)\\ & \;\;\;+\left(ZG_{t}+\frac{1}{2}\left(\theta -\beta \right)n_{t}\sigma^{2}+\left(1-M\right)Rw_{t+1}n_{t}\sigma^{2}\right)U_{2}\left(t\right). \end{array}$$

We next show the scheduling scheme for each time slot. Putting the value of the parameters θ and β back to (20), we get the following:

(21) $$\begin{array}{cc} \; & \min_{\pi_{t}}\;\left[VH_{t}-\left(w_{t+1}-\tilde{w}+\frac{\tilde{w}}{p\varphi_{1}}\right)Rp{Q_{t}}^{2}\right]U_{1}\left(t\right)\\ & \;\;\;+\left[ZG_{t}+\left(\left(M-1\right)\left(\tilde{w}-w_{t+1}\right)-\frac{\tilde{w}M}{\varphi_{2}}\right)Rn_{t}\sigma^{2}\right]U_{2}\left(t\right). \end{array}$$

Set the update index $a_{t}=VH_{t}-\left(w_{t+1}-\tilde{w}+\frac{\tilde{w}}{p\varphi_{1}}\right)Rp{Q_{t}}^{2}$ and update index $b_{t}=ZG_{t}+\left(\left(M-1\right)\left(\tilde{w}-w_{t+1}\right)-\frac{\tilde{w}M}{\varphi_{2}}\right)Rn_{t}\sigma^{2}$, and then the solution to the scheme (20) can be achieved:

U1(t)={(22a)1 ,at<0(22b)0 ,otherwise

U2(t)={(23a)1 ,bt<0(23b)0 ,otherwise.

We summarize below the resulting Lyapunov optimal Algorithm 1.
**Algorithm 1** Decisions scheduling scheme based on Lyapunov optimization**Input:**$A_{0},Q_{0},H_{0},G_{0},n_{0},S_{t},K_{t},\varphi_{1},\varphi_{2},w_{t},\tilde{w},V,Z,M,R$ 1:**for** each time slot *t* **do** 2:   Calculate $a_{t}=VH_{t}-\left(w_{t+1}-\tilde{w}+\frac{\tilde{w}}{p\varphi_{1}}\right)Rp{Q_{t}}^{2}$; 3:   Calculate $b_{t}=ZG_{t}+\left(\left(M-1\right)\left(\tilde{w}-w_{t+1}\right)-\frac{\tilde{w}M}{\varphi_{2}}\right)Rn_{t}\sigma^{2}$; 4:   **if** $a_{t}<0$ **then** 5:     $U_{1}\left(t\right)=1$ 6:   **else** 7:     $U_{1}\left(t\right)=0$; 8:   **end if** 9:   **if** $b_{t}<0$ **then**10:     $U_{2}\left(t\right)=1$;11:   **else**12:     $U_{2}\left(t\right)=0$;13:   **end if**14:   Calculate $A_{t+1}=\left(1-U_{2}\left(t\right)\right)A_{t}+K_{t}$;15:   Calculate $Q_{t+1}=\left(1-S_{t}U_{1}\left(t\right)\right)Q_{t}+U_{2}\left(t\right)A_{t}$;16:   Calculate $H_{t+1}=\max\left\{H_{t}-\varphi_{1}+U_{1}\left(t\right),\;0\right\}$;17:   Calculate $G_{t+1}=\max\left\{G_{t}-\varphi_{2}+U_{2}\left(t\right),\;0\right\}$;18:   Calculate $n_{t+1}=\left(1-U_{2}\left(t\right)\right)n_{t}+1$;19:**end for**

Based on the algorithm, we can make decisions by scheduling every time slot to minimize the value of UoI and maintain the virtual queue stability simultaneously. From the algorithm, it is apparent that we can successfully decouple the joint decisions into two independent threshold schemes, which makes the implementation desirably simple.

### 3.4. Solving for the Target Function and Lyapunov Gap

In this section, we will solve for the target function and achieve the expression of the gap between the optimal solution and the result obtained by the Lyapunov optimization algorithm. We will also prove that the result gained by the Lyapunov optimization algorithm can be infinitely close to the optimal solution. Now make the summation of the total T-time slot on both sides of (19), and we can get

(24) $$\begin{array}{c} E\left[L_{T}-L_{0}+\sum_{t=0}^{T-1}f_{t}\right]\leq T\left[\left(\frac{1}{p\varphi_{1}}+\frac{M}{\varphi_{2}}\right)R\tilde{w}\sigma^{2}+\frac{1}{2}\left(V+Z\right)\right]. \end{array}$$

Note that $L_{T}\geq 0$ and $\frac{L_{0}}{T}=0$, and then divide *T* on both sides of (24) to get the time-averaged result

(25) $$\begin{array}{c} \frac{1}{T}E\left[\sum_{t=0}^{T-1}f_{t}\right]\leq \left(\frac{1}{p\varphi_{1}}+\frac{M}{\varphi_{2}}\right)R\tilde{w}\sigma^{2}+\frac{1}{2}\left(V+Z\right). \end{array}$$

**Theorem** **1.** *Set the problem of (20) as $P_{2}\left(\pi_{t}\right)$, and then the solution of $P_{2}\left(\pi_{t}\right)$ will satisfy the following gap:*

(26) $$\begin{array}{c} \frac{1}{T}\sum_{t=0}^{T-1}E\left[w_{t}({Q_{t}}^{2}+M{A_{t}}^{2})\right]\leq \left(\frac{1}{p\varphi_{1}}+\frac{M}{\varphi_{2}}\right)\tilde{w}\sigma^{2}+\frac{\left(V+Z\right)}{2R} \end{array}$$

*That is, the solution of $P_{2}\left(\pi_{t}\right)$ can be approximated by the solution of $P_{1}\left(\pi_{t}\right)$, and the gap between them is $\frac{\left(V+Z\right)}{2R}$.*

**Proof.**  See Appendix D.      □

To be precise, the proof of this gap result in Appendix D requires $A_{t}$ and $Q_{t}$ in (2) and (3) to be lower bounded by zero. Nevertheless, our numerical results show consistence with this gap even when they are non-negative. Note that as the value of *R* is taken as large as possible, and the result obtained by the Lyapunov optimization algorithm $P_{2}\left(\pi_{t}\right)$ can be made arbitrarily close to the optimal result $P_{1}^{*}\left(\pi_{t}\right)$. $\frac{\left(V+Z\right)}{2R}$ can also seem to be the ratio of the weight of the energy constraints and UoI, which shows the tradeoff between the UoI and the energy constraints.

## 4. Numerical Results

In this section, we present extensive numerical results to explore the behaviour of the optimal scheme under various constraints and scenarios. At the beginning of each time slot, the terminal first decides whether to transmit the error packets to the service center or not. The transmission takes 1 ms and goes through a wireless channel with a fixed failure transmission rate. At the same time, the service center transmits the estimation error (feedback) to the terminal, which also takes 1 ms with no failure rate. At the end of each time slot, the feedback arrives at the terminal, and the terminal will decide whether to sense this feedback. Meanwhile, the service channel receives the error packets and the latest estimation of Gaussian noise. The service center will immediately calculate the error difference between the transmitted status information and the received status information and add that new error into the error packet.

### 4.1. Response to Urgency Levels

**Figure 2:**
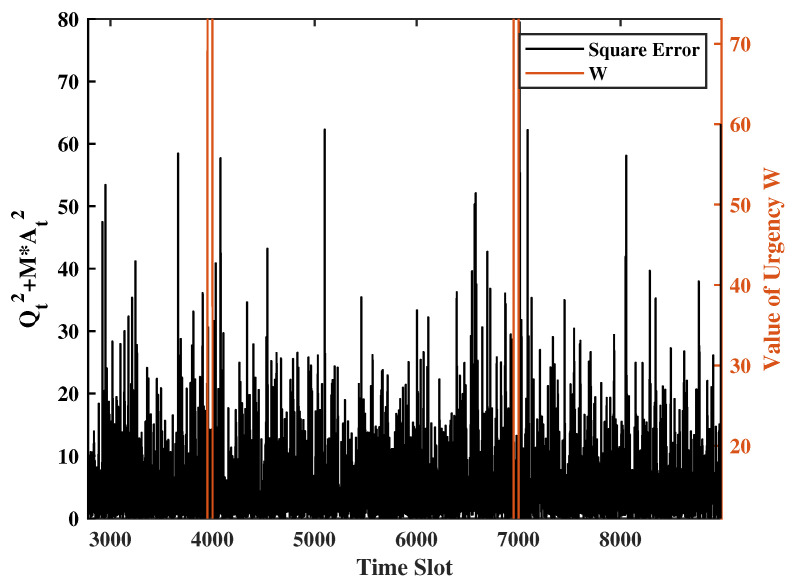
UoI sequence obtained by the proposed Lyapunov algorithm under a specific realization of weights $w_{t}$.

**Figure 3:**
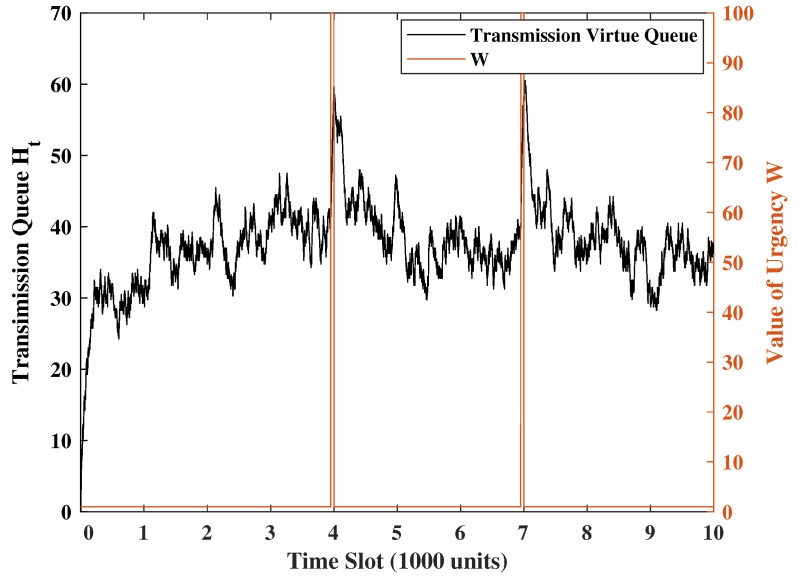
Transmission virtual queue under the same realization of weight $w_{t}$ in Figure 2.

**Figure 4:**
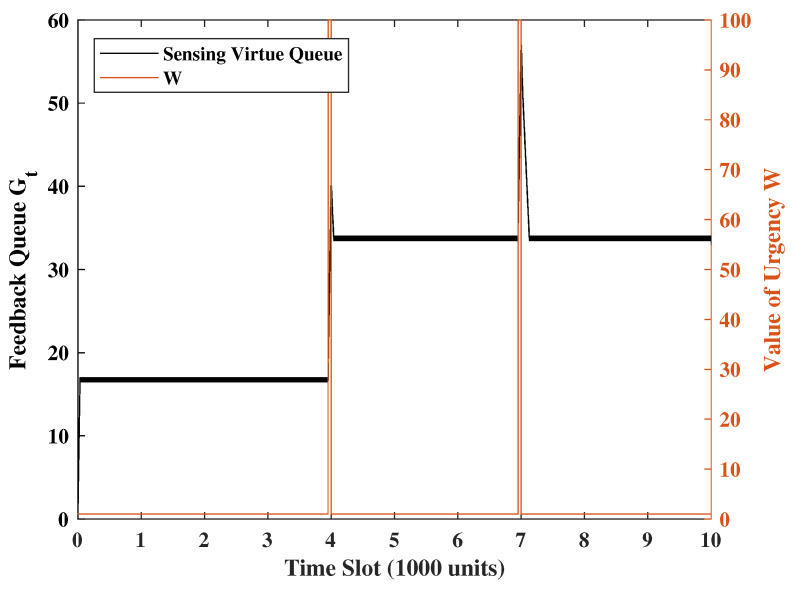
Sensing virtual queue under the same realization of weight $w_{t}$ in Figure 2.

To demonstrate the system’s response to a new urgency, for every 5000 time slots, we set W = 100 in the 50 consecutive time slots and W = 1 in the rest of the time slots. The transmission/sense energy constraints are set as $\varphi_{1}=0.25$ and $\varphi_{2}=0.5$. The channel error rate is p=0.8, the weight of the UoI is set as M=2.5 and R=2, and the weight of the system states is set as V=Z=1. Additionally, the Gaussian noise variance will be set to unity. Figure 2, Figure 3 and Figure 4 show a sample evolution of the squared of errors $MA_{t}^{2}+Q_{t}^{2}$ and two virtual queue length $H_{t},G_{t}$. Observing Figure 2, Figure 3 and Figure 4, we understand that when the urgency level rises, the square of errors will drop significantly, and the virtual queues will keep increasing because update transmissions are ramped up. However, due to the energy constraints, the terminal’s probability of transmitting and sensing are affected. This is the reason why the square of errors will increase, and the transmission virtual queue will decrease after the urgency. These show that the system can swiftly respond to urgency levels while keeping the error variance portion of UoI (i.e., $Q_{t}^{2}+MA_{t}^{2}$) at a reasonable level at all times.

### 4.2. Tradeoff between UoI and System Parameters

In this section, we compare how the relationship between different variables will affect the UoI in the system. Unless otherwise specified, we set the energy constraint of transmission/sensing as $\varphi_{1}=\varphi_{2}=0.8$, the weight of the system stability as V=Z=1, the weight of totally UoI and the UoI in service center as R=M=2, the channel error rate as p=0.8, and the weight of urgency at each time slot is i.i.d. with probability 0.99 being 1 and probability 0.01 being 100.

**Figure 5:**
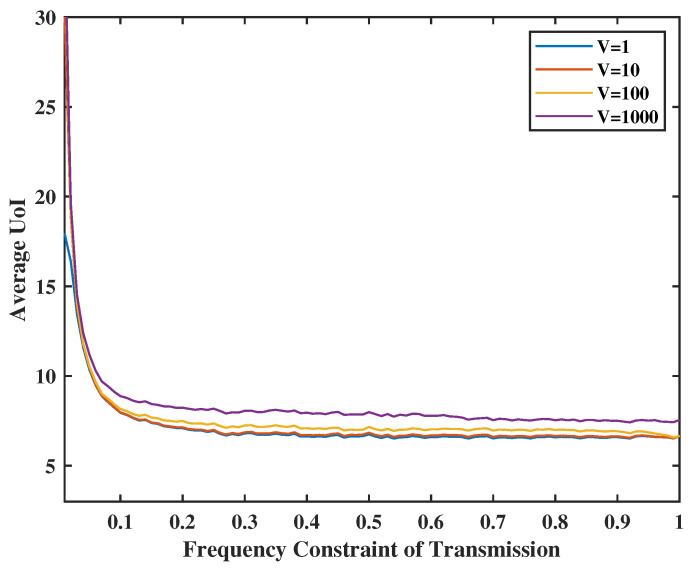
Tradeoff between transmission energy constraint, V and UoI.

**Figure 6:**
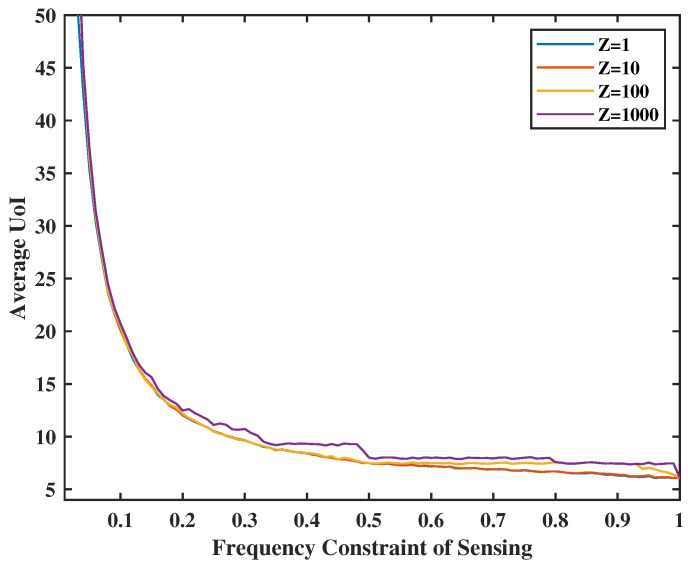
Tradeoff between sense energy constraint, Z and UoI.

Figure 5 and Figure 6 present the relationship between UoI and transmission/sense energy constraint. They also show the effect of system stability weights on UoI. In Figure 5, the energy constraint of transmission ranges from 0.1 to 1.0, and the weight of the queue stability (i.e., the virtual queue levels) in the transmission part will be set as *V* = 1, 10, 100, and 1000. Similarly, in Figure 6, we set the energy constraint of sensing from 0.1 to 1.0 and *Z* = 1, 10, 100, and 1000. We observe that when average energy is less constrained, the UoI decreases. However, the UoI will not change much when the transmission frequency reaches 0.5. This is due to the fact that the frequency constraint becomes inactive after a certain level depending on the sensing activity. As sensing and transmission are in tandem, the higher frequency drives the overall performance. Moreover, when the weight of the stability *V* and *Z* are small, e.g., V=1 or Z=1, we pay more attention to the value of UoI than the frequency of transmission levels, yielding a virtual queue significantly above the set constraint. On the other hand, if we set the weight of the stability *V* and *Z* at a high level, e.g., V=1000 or Z=1000, the virtual queue stability becomes much more important, which compromises UoI performance.

**Figure 7:**
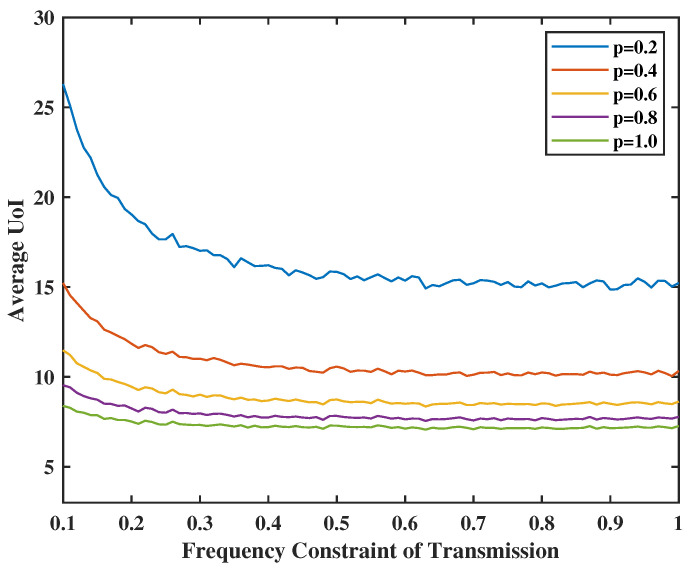
Tradeoff between transmission energy constraint, channel failure rate *p*, and UoI.

**Figure 8:**
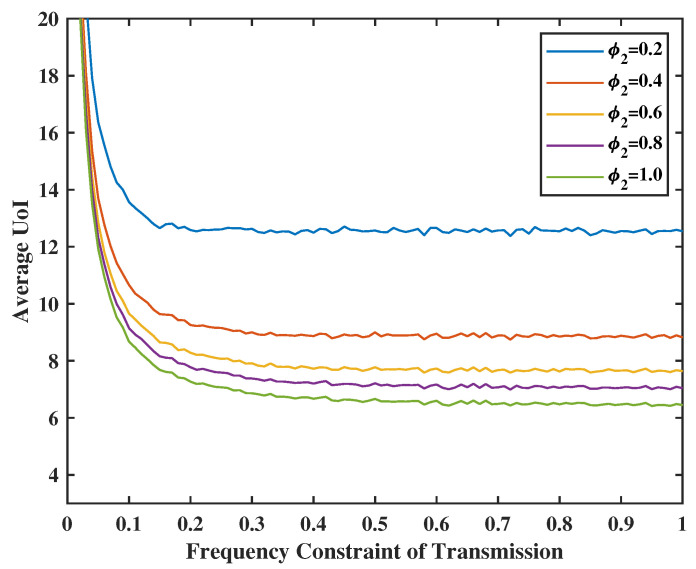
Tradeoff between transmission energy constraint, sensing energy constraint, and UoI.

In Figure 7, the energy constraint of transmission will be set from 0.1 to 1.0, and the failure probability of transmission will be set as p=0.2,0.4,0.6,0.8, and 1.0. We observe that the higher *p* is, the lower the average UoI is. This is because we need to decide to transmit more frequently to achieve the optimal average of UoI when the success rate is lower. In Figure 8, we observe that the average UoI decreases no matter whether $\varphi_{1}$ or $\varphi_{2}$ increases because we have more chances to transmit or sense when the energy is sufficient. Additionally, as $\varphi_{2}$ gets smaller, the curve will converge earlier because the error packets in the service center are the input of the terminal. When we have less probability of sensing the feedback, the transmission frequency will also not be large because of the input limitation, even if the transmission energy is sufficient.

**Figure 9:**
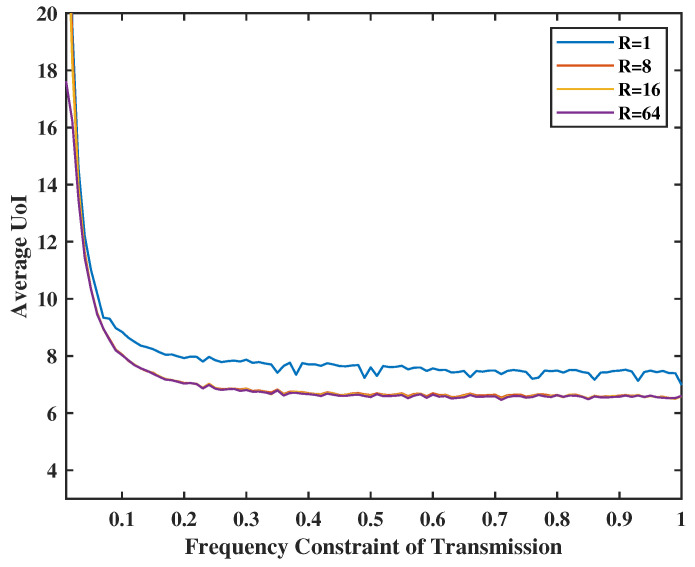
Tradeoff between transmission energy constraint, R and UoI.

In Figure 9, we set the weight of total UoI as R=1,8,16 and 64, and the weight of two virtual queues as V=Z=20. As expected, the larger the weight of the total UoI is, the smaller the average UoI will be. This is because the system will consider the UoI more important and will take more chances to transmit and sense. Moreover, the Lyapunov gap, i.e., $\frac{\left(V+Z\right)}{2R}$, will diminish as R increases.

### 4.3. Tradeoff between UoI and System Stability

The tradeoff between the target function and the system stability is always an exciting and crucial question in the Lyapunov optimization framework. This section will show examples of how different weights can affect the system stability and UoI. We set T=10,000 and channel error rate as p=0.8. The urgency weight $w_{t}$ is determined as an i.i.d. random process with probability 0.99 being 1 and probability 0.01 being 1000. We will observe the number of update transmissions and senses (i.e. the energies spent for update transmission and sensing throughout T=10,000 slots) to represent system stability.

**Figure 10:**
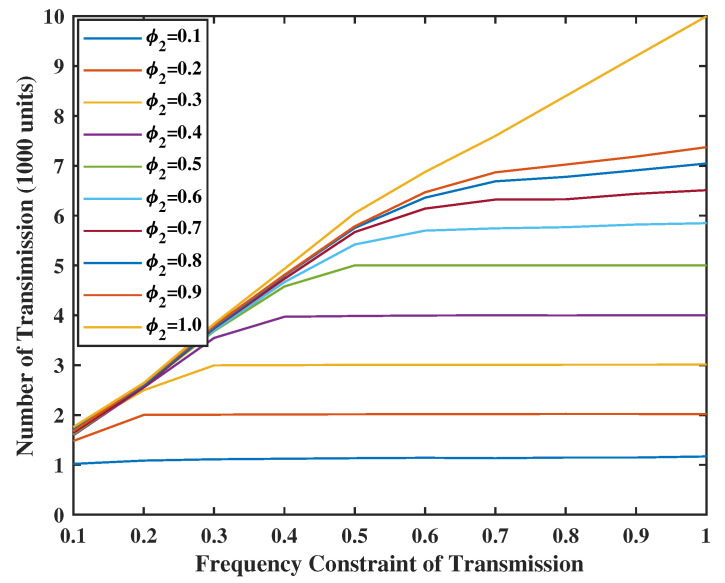
Energy spent for update transmission when V = Z = 10.

**Figure 11:**
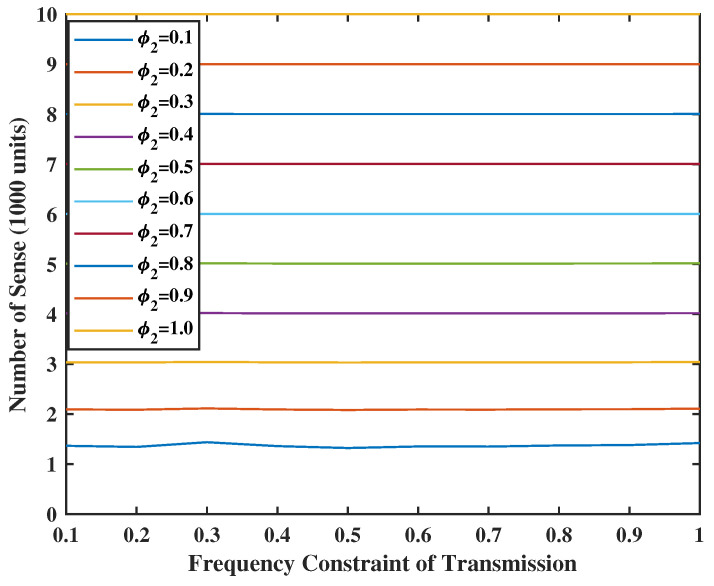
Energy spent for sensing when V = Z = 10.

In Figure 10 and Figure 11, we set the weight of the system stability as V=Z=10, and the weight of UoI as R=M=2. As the energy is sufficient, we can have more chances to transmit and sense. In addition, the number of transmissions is always smaller or equal to the number of senses. This makes sense because the input error in the terminal comes from the service center and will be sent together in one transmission. In addition, even if there is no energy constraint for the transmission, e.g., $\varphi_{1}=1.0$, the number of transmissions will not reach the value of constraints. This is due to the fact that the frequency constraint becomes inactive after a certain level. However, when $\varphi_{2}\leq 0.2$, the energy spent for sensing goes above the set energy constraints. The reason is that the weight of UoI is much larger than the weight of stability. This means that the system will sacrifice stability for better UoI.

**Figure 12:**
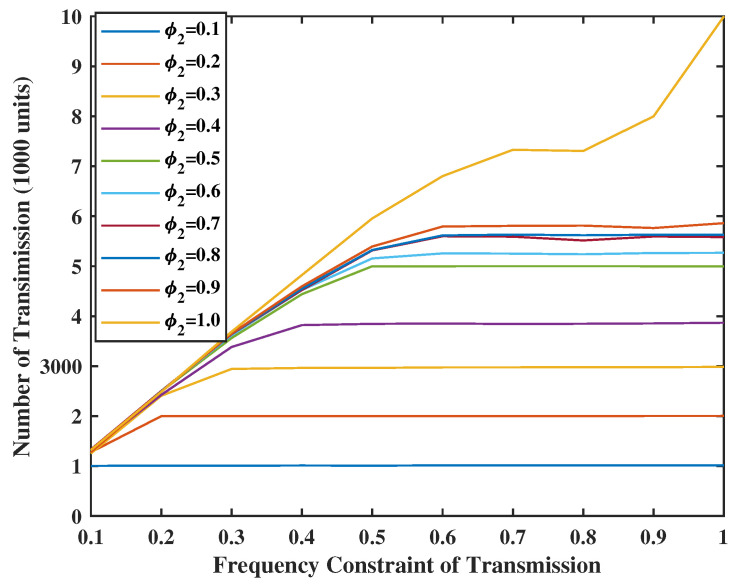
Energy spent for update transmission when V = Z = 80.

**Figure 13:**
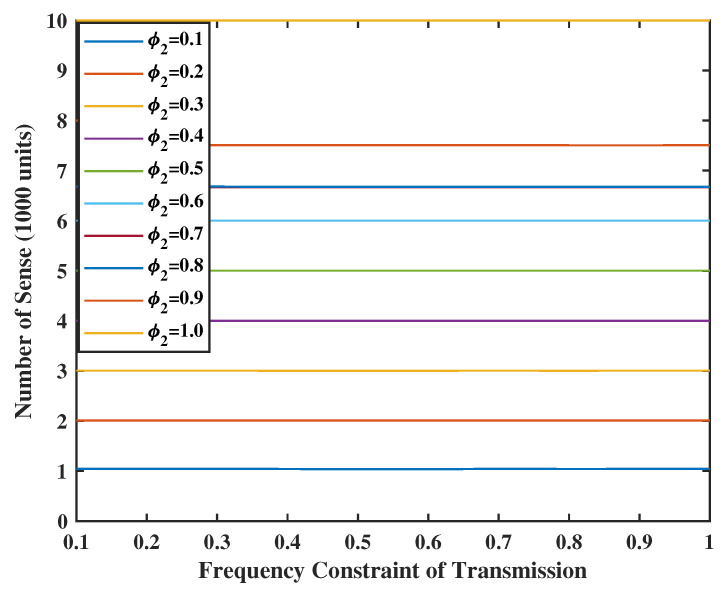
Energy spent for sensing when V = Z = 80.

In Figure 12 and Figure 13, we set the weight of the system stability as V=Z=80, which is larger than the weight of UoI. We see that both the transmission and sensing constraints are not binding. Comparing with the Figure 5 and Figure 6, we observe that the UoI with V,Z=100 is close to the UoI with V,Z=1. Hence, by sacrificing a small amount of UoI, a very stable system can be guaranteed.

**Figure 14:**
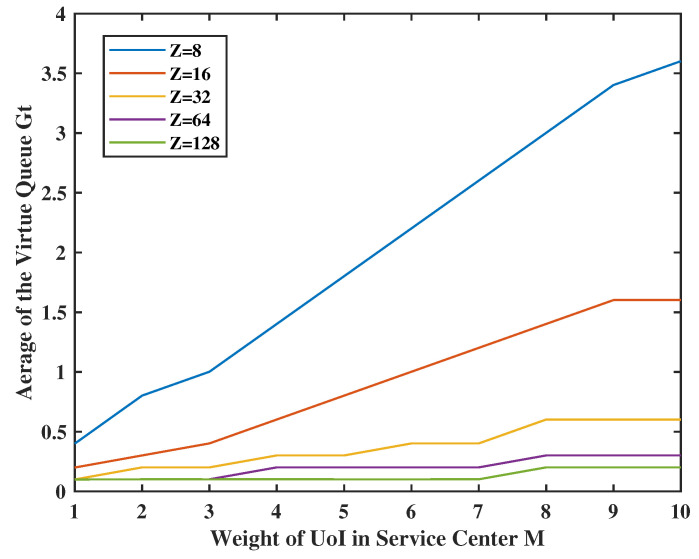
Tradeoff between M,Z and sensing frequency.

In Figure 14, we set the weight of the UoI in service center as M∈[1,10], the weights Z = 8, 16, 32, 64, and 128, and V=5, $\varphi_{1}=0.5$ and $\varphi_{2}=0.8$. The virtual queue $G_{t}$ is small when its weight is large, and the energy constraints are tight. In addition, when the weight of UoI in the service center *M* increases, the sensing time will keep increasing because the UoI in the service center is much more important than the virtual queue stability and the information in the terminal. This also exemplifies that our framework can accommodate different cases flexibly by using different weights.

### 4.4. Comparison of Lyapunov Optimal Performance with Other Algorithms

**Figure 15:**
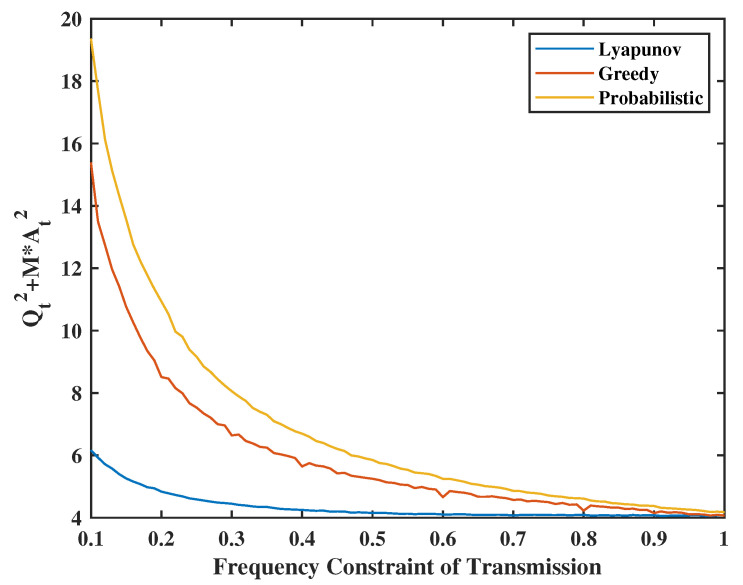
Lyapunov optimal algorithm and greedy algorithm.

The greedy and probabilistic algorithms are also very suitable naive algorithms to solve this problem. The main idea of the greedy algorithm is that the terminal will decide to transmit/sense at time *t* if the instantaneous transmission/sensing frequency at time *t* has not reached the corresponding set limits. Moreover, for the probabilistic algorithm, in each time slot the terminal will transmit/sense with probability equal to the value of frequency constraints. For Figure 15, we set the weight of system stability as V=Z=30. Channel success rate is set as p=0.6, sense energy constraint is set as $\varphi_{2}=0.8$, and the weight of urgency at each time slot is the same as before. Because the greedy algorithm takes action independent of urgency, we will compare the average UoI with $w_{t}=1$. From the figure, the average error portion of UoI (i.e., $Q_{t}^{2}+MA_{t}^{2}$) obtained by Lyapunov optimization is always lower than the other two algorithms, especially when the energy is insufficient and the gap closes with increasing energy availability.

Recall that UoI can subsume various AoI problems. For instance, if we set the cost function δ(.) as a linear function with the unit parameter and the urgency weight $w_{t}=1$, then we can express the AoI in terminal ${\tilde{Q}}_{t}$ and the AoI in service center ${\tilde{A}}_{t}$ as

(27) $$\begin{array}{cc} {\tilde{A}}_{t+1} & =\left(1-U_{2}\left(t\right)\right){\tilde{A}}_{t}+1 \end{array}$$

(28) $$\begin{array}{cc} {\tilde{Q}}_{t+1} & =\left(1-S\left(t\right)U_{1}\left(t\right)\right){\tilde{Q}}_{t}+U_{2}\left(t\right){\tilde{A}}_{t}. \end{array}$$

**Figure 16:**
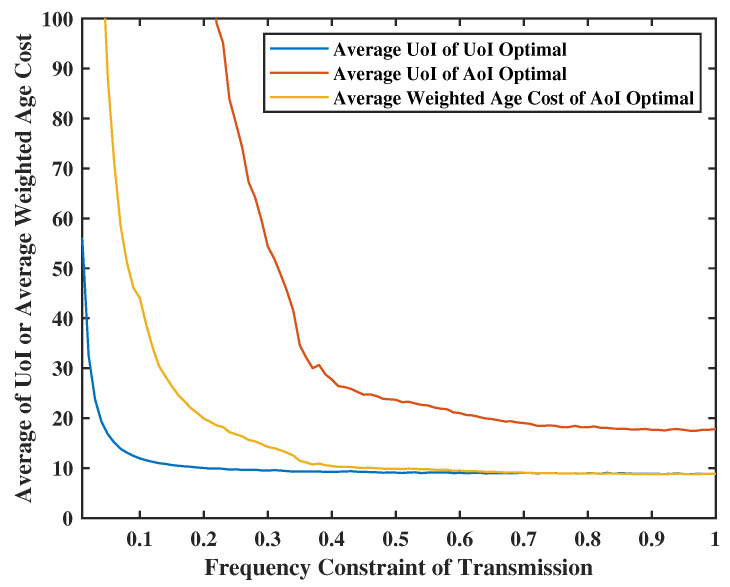
Lyapunov optimization vs. AoI optimal.

Let us use the same Lyapunov optimization algorithm described earlier along with the same weights for system state variables and target function for a fair comparison. In Figure 16, we set the weight of virtual queues as V=Z=20, the weight of UoI as R=M=2, the weight of system states θ,β in AoI optimal will be the same as the value of UoI optimal and will be calculated each round. Additionally, set the probability of fail transmission as p=0.6, sensing frequency limitation as $\varphi_{2}=0.6$. We can deduce that the average UoI obtained by UoI optimal is much better than the value obtained by AoI optimal. In addition, the value of average UoI by UoI optimal is smaller than that of the average weighted AoI by AoI optimal. This is because, in the AoI model, the increment $K_{t}$ will always be 1; however, the UoI model yields a lower expectation.

## 5. Conclusions

This paper focused on urgency of information (UoI) optimization through joint sensing and transmission. We proposed a new interactive status updating problem over a point-to-point channel in which transmission and sensing actions are determined to minimize UoI as a combination of the staleness of sensed data and synchronization between two ends under resource constraints, and we used a Lyapunov optimization framework for its optimization. We obtained the gap between the optimal solution and the result gained by the Lyapunov optimal algorithm, and proved that the gap between them can be made arbitrarily small. We presented an extensive numerical study that illustrates various features of the model and resulting algorithm, and potential performance improvements with respect to several schemes. In our future work, we plan to extend this work in multiple directions such as the case of multiple terminals in series or parallel, on demand UoI definition and optimization as well as the cases of computation transmission tradeoffs and dynamical energy constraints.

## Figures and Tables

**Table 1:** Definitions of Variables.

Symbol	Description
$A_{t}$	Error in Service Center
$Q_{t}$	Error in Terminal
$H_{t}$	Transmission Frequency Virtual Queue
$G_{t}$	Sensing Frequency Virtual Queue
$n_{t}$	Number of Time Slots since Last Sense
$S_{t}$	Channel Situation
$U_{1}\left(t\right),U_{2}\left(t\right)$	Transmission/Sensing Decision
$\pi_{t}$	Set of Decisions
$\varphi_{1},\varphi_{2}$	Transmission/Sensing Frequency Constraints
$w_{t}$	Weight of Urgency
$\tilde{w}$	Average Weight of Urgency
V,Z,θ,β	Weight of $H_{t}^{2},G_{t}^{2},Q_{t}^{2},A_{t}^{2}$ in Drift Function
*M*	Weight of $A_{t}^{2}$ in Target Function
*R*	Weight of Penalty Compared with Drift
$L_{t}$	Summation of all the Queues
$\Delta_{t}$	Lyapunov Drift
$f_{t}$	Lyapunov Penalty
$Y_{t}$	Set of Given Parameters in $t_{th}$ Time Slot

## Data Availability

Not applicable.

## Appendix A

Based on (7) we can get the following sequence of steps:

(A1) $$\begin{array}{cc} \; & E\left[H_{t+1}^{2}-{H_{t}}^{2}|Y_{t}\right]\\ \; & \leq E\left[{\left(H_{t}-\varphi_{1}+U_{1}\left(t\right)\right)}^{2}-{H_{t}}^{2}|Y_{t}\right]\\ \; & =E\left[H_{t}^{2}+\varphi_{1}^{2}+U_{1}{\left(t\right)}^{2}-2\varphi_{1}H_{t}+2H_{t}U_{1}\left(t\right)-2\varphi_{1}U_{1}\left(t\right)-H_{t}^{2}|Y_{t}\right]\\ \; & =E\left[{\left(\varphi_{1}-U_{1}\left(t\right)\right)}^{2}+2\left(-\varphi_{1}+U_{1}\left(t\right)\right)H_{t}|Y_{t}\right]\\ \; & \leq 1+2\left(-\varphi_{1}+E\left[U_{1}\left(t\right)|Y_{t}\right]\right)H_{t}, \end{array}$$

where the first inequality follows from the definition of $H_{t}$ in (7) used in the identity that for any $X=\max\left\{a+b-c,0\right\}$, $X^{2}\leq {\left(a+b-c\right)}^{2}$, the following equalities follow from rearranging terms and the final inequality follow from $\varphi_{1}-U_{1}\left(t\right)\leq 1$. Based on Equation (8), and using the same method as that for obtaining (A1), we get the following inequality:

(A2) $$\begin{array}{c} E\left[{G_{t+1}}^{2}-{G_{t}}^{2}|Y_{t}\right]\leq 1+2\left(-\varphi_{2}+E\left[U_{2}\left(t\right)|Y_{t}\right]\right)G_{t}. \end{array}$$

Based on (2), we have

(A3) $$\begin{array}{cc} \; & E\left[{A_{t+1}}^{2}-{A_{t}}^{2}|Y_{t}\right]\\ \; & =E\left[{\left(1-U_{2}\left(t\right)\right)}^{2}{A_{t}}^{2}+2A_{t}K_{t}\left(1-U_{2}\left(t\right)\right)+{K_{t}}^{2}-{A_{t}}^{2}|Y_{t}\right]. \end{array}$$

Recall that $K_{t}∼\left(0,\sigma^{2}\right)$ follows i.i.d Gaussian distributions. This is due to the fact that the queue $A_{t}$ is the summation of $K_{t}$; it is obvious that the summation of the Gaussian distribution is still a Gaussian distribution. As a result, the error in the service center also follows a Gaussian distribution $A_{t}∼\left(0,n\sigma^{2}\right)$. In addition, as $U_{2}\left(t\right)\in \left\{0,1\right\}$, we can simplified (A3) by $U_{2}{\left(t\right)}^{2}=U_{2}\left(t\right)$ and ${\left(1-U_{2}\left(t\right)\right)}^{2}=1-U_{2}\left(t\right)$. As a result, we have

(A4) $$\begin{array}{c} E\left[{A_{t+1}}^{2}-{A_{t}}^{2}|Y_{t}\right]=-{n_{t}\sigma }^{2}E\left[U_{2}\left(t\right)|Y_{t}\right]+\sigma^{2}. \end{array}$$

Based on (3) and the fact that ${\left(1-U_{1}\left(t\right)S_{t}\right)}^{2}=\left(1-U_{1}\left(t\right)S_{t}\right)$, we have

(A5) $$\begin{array}{cc} \; & E\left[{Q_{t+1}}^{2}-{Q_{t}}^{2}|Y_{t}\right]\\ \; & =E\left[{\left(1-U_{1}\left(t\right)S_{t}\right)}^{2}{Q_{t}}^{2}+2Q_{t}A_{t}U_{2}\left(t\right)\left(1-U_{1}\left(t\right)S_{t}\right)+{A_{t}}^{2}{U_{2}\left(t\right)}^{2}-{Q_{t}}^{2}|Y_{t}\right]\\ \; & =-{Q_{t}}^{2}pE\left[U_{1}\left(t\right)|Y_{t}\right]+{n_{t}\sigma }^{2}E\left[U_{2}\left(t\right)|Y_{t}\right]. \end{array}$$

Based on (A1)–(A5), we have

(A6) $$\begin{array}{cc} \Delta_{t} & =E\left[L_{t+1}-L_{t}|Y_{t}\right]\\ \; & =E\left[\frac{1}{2}V\left(H_{t+1}^{2}-{H_{t}}^{2}\right)+\frac{1}{2}Z\left(G_{t+1}^{2}-G_{t}^{2}\right)+\frac{1}{2}\theta \left(Q_{t+1}^{2}-Q_{t}^{2}\right)+\frac{1}{2}\beta \left(A_{t+1}^{2}-{A_{t}}^{2}\right)|Y_{t}\right]\\ \; & \leq \frac{1}{2}\left(V+Z\right)+\frac{1}{2}\beta \sigma^{2}-V\varphi_{1}H_{t}-Z\varphi_{2}G_{t}+\left(VH_{t}-\frac{1}{2}\theta p{Q_{t}}^{2}\right)E\left[U_{1}\left(t\right)|Y_{t}\right]\\ \; & +\left(ZG_{t}+\frac{1}{2}\theta n_{t}\sigma^{2}-\frac{1}{2}\beta n_{t}\sigma^{2}\right)E\left[U_{2}\left(t\right)|Y_{t}\right]. \end{array}$$

## Appendix B

Set the Lyapunov penalty function as $f_{t}=Rw_{t+1}({Q_{t+1}}^{2}+M{A_{t+1}}^{2})$, where $w_{t}\geq 0$. Based on (A4) and (A5), we can get the Lyapunov penalty function as follows:

(A7) $$\begin{array}{cc} E\left[f_{t}|Y_{t}\right]= & RE\left[w_{t+1}\left(-Q_{t}^{2}S_{t}U_{1}\left(t\right)+2Q_{t}A_{t}U_{2}\left(t\right)\left(1-S_{t}U_{1}\left(t\right)\right)+A_{t}^{2}U_{2}\left(t\right)+Q_{t}^{2}\right)|Y_{t}\right]\\ \; & +RME\left[w_{t+1}\left(-A_{t}^{2}U_{2}\left(t\right)+K_{t}^{2}+A_{t}^{2}\right|Y_{t}\right]\\ = & R\tilde{w}\left(-Q_{t}^{2}pE\left[U_{1}\left(t\right)|Y_{t}\right]+Q_{t}^{2}+M\sigma^{2}+Mn_{t}\sigma^{2}+\left(1-M\right)n_{t}\sigma^{2}E\left[U_{2}\left(t\right)|Y_{t}\right]\right). \end{array}$$

Combining (A6) and (A7), the Lyapunov drift plus penalty function satisfies the following inequality:

(A8) $$\begin{array}{cc} \Delta_{t}+E\left[f_{t}|Y_{t}\right]\leq & \frac{1}{2}\left(V+Z\right)+\frac{1}{2}\beta \sigma^{2}+R\tilde{w}\left(Q_{t}^{2}+M\sigma^{2}+Mn_{t}\sigma^{2}\right)-V\varphi_{1}H_{t}-Z\varphi_{2}G_{t}\\ \; & +\left(VH_{t}-\frac{1}{2}\theta p{Q_{t}}^{2}-R\tilde{w}pQ_{t}^{2}\right)E\left[U_{1}\left(t\right)|Y_{t}\right]\\ \; & +\left(ZG_{t}+\frac{1}{2}\theta n_{t}\sigma^{2}-\frac{1}{2}\beta n_{t}\sigma^{2}+\left(1-M\right)R\tilde{w}n_{t}\sigma^{2}\right)E\left[U_{2}\left(t\right)|Y_{t}\right]. \end{array}$$

## Appendix C

We start by assuming that the initial values satisfy $E\left[L_{0}\right]<\infty$. If $\Delta_{t}+E\left[f_{t}\right]\leq C$, where *C* is a constant, then take the summation over *T* time slots to get

(A9) $$\begin{array}{c} E\left[L_{T}-L_{0}+\sum_{t=0}^{T-1}f_{t}\right]\leq TC. \end{array}$$

Based on (9) we have

(A10) $$\begin{array}{c} E\left[L_{T}\right]\geq \frac{1}{2}VE\left[(H_{t}^{2})\right]. \end{array}$$

From the definition of the virtual queue $H_{t}$ (A1), it is obviously that $E\left[(H_{t}^{2})\right]\;\geq {\left(E\left[(H_{t})\right]\;\right)}^{2}$, and also because the penalty function $f_{t}$ is always non-negative, we can change (A9) into

(A11) $$\begin{array}{c} \frac{1}{2}VE\left[(H_{t})\right]{)}^{2}\leq TC+L_{0}\\ E\left[(H_{t})\right]\leq \frac{\sqrt{2(TC+L_{0})}}{V}\\ \frac{E\left[(H_{t})\right]}{T}\leq \frac{\sqrt{2(TC+L_{0})}}{VT}. \end{array}$$

Since T→∞, the right hand side of (A11) is equal to 0. As a result,

(A12) $$\begin{array}{c} \frac{E\left[(H_{t})\right]}{T}\to 0. \end{array}$$

As a result, the virtual queue $H_{t}$ is mean rate stable. The other system states $Q_{t},A_{t}$ and the virtual queue $Z_{t}$ can be proven as mean rate stable with the same method above. Therefore, the expressions of the queues in the system are appropriate, and the Lyapunov optimization algorithm is applicable. We also recall that the evolution of $Q_{t},A_{t}$, although not originally in a queue form, can be easily redefined to be bounded below by 0, and the analysis in our paper will be valid without any changes.

## Appendix D

First, let us assume that $P_{1}$ has an optimal solution, which is to take the best decision for every time slot and get the optimal result of the target function (4). Because this optimal solution does not use the Lyapunov algorithm, the decision has no relationship with the queues and virtual queues in the system. Below $\pi_{t}=\left\{U_{1}\left(t\right),U_{2}\left(t\right)\right\}$ will be used to represent the decision policy of the Lyapunov optimization algorithm and $\pi_{t}^{*}=\left\{U_{1}{\left(t\right)}^{*},U_{2}^{*}\left(t\right)\right\}$ is used to represent the decisions of the optimal solution. Based on Equations (A1)–(A5), we have

(A13) $$\begin{array}{c} L_{t+1}-L_{t}+f_{t}\left(\pi_{t}\right)\leq \frac{1}{2}\left(V+Z\right)+\left(-\varphi_{1}+U_{1}\left(t\right)\right)H_{t}+\left(-\varphi_{2}+U_{2}\left(t\right)\right)G_{t}\\ +\frac{1}{2}\theta \left(-{Q_{t}}^{2}U_{1}\left(t\right)S_{t}+2Q_{t}A_{t}U_{2}\left(t\right)\left(1-U_{1}\left(t\right)S_{t}\right)+{A_{t}}^{2}U_{2}\left(t\right)\right)\\ +\frac{1}{2}\beta \left(-U_{2}\left(t\right){A_{t}}^{2}+2A_{t}K_{t}\left(1-U_{2}\left(t\right)\right)+{K_{t}}^{2}\right)+f_{t}\left(\pi_{t}\right). \end{array}$$

Because the optimal solution is a solution of the problem, it should also obey (A13)

(A14) $$\begin{array}{c} L_{t+1}-L_{t}+f_{t}\left(\pi_{t}\right)\leq \frac{1}{2}\left(V+Z\right)+\left(-\varphi_{1}+U_{1}^{*}\left(t\right)\right)H_{t}+\left(-\varphi_{2}+U_{2}^{*}\left(t\right)\right)G_{t}\\ +\frac{1}{2}\theta \left(-{Q_{t}}^{2}U_{1}^{*}\left(t\right)S_{t}+2Q_{t}A_{t}U_{2}^{*}\left(t\right)\left(1-U_{1}^{*}\left(t\right)S_{t}\right)+{A_{t}}^{2}U_{2}^{*}\left(t\right)\right)\\ +\frac{1}{2}\beta \left(-U_{2}^{*}\left(t\right){A_{t}}^{2}+2A_{t}K_{t}\left(1-U_{2}^{*}\left(t\right)\right)+{K_{t}}^{2}\right)+f_{t}\left(\pi_{t}^{*}\right). \end{array}$$

Then take the expectation on both sides of (A14)

(A15) $$\begin{array}{c} E\left[L_{t+1}-L_{t}+f_{t}\left(\pi_{t}\right)|Y_{t}\right]\leq \frac{1}{2}\left(V+Z\right)+E\left[\left(-\varphi_{1}+U_{1}^{*}\left(t\right)\right)H_{t}\right]+E\left[\left(-\varphi_{2}+U_{2}^{*}\left(t\right)\right)G_{t}\right]\\ +\frac{1}{2}\theta E\left[-{Q_{t}}^{2}U_{1}^{*}\left(t\right)S_{t}+2Q_{t}A_{t}U_{2}^{*}\left(t\right)\left(1-U_{1}^{*}\left(t\right)S_{t}\right)+{A_{t}}^{2}U_{2}^{*}\left(t\right)\right]\\ +\frac{1}{2}\beta E\left[-U_{2}^{*}\left(t\right){A_{t}}^{2}+2A_{t}K_{t}\left(1-U_{2}^{*}\left(t\right)\right)+{K_{t}}^{2}\right]+E\left[f_{t}\left(\pi_{t}^{*}\right)\right]. \end{array}$$

As is well known in the literature [7,8], there exists a w-optimal decision rule that makes decision randomly and independent of the variables in the system. In the analysis below, we assume such an optimal policy and denote it as $\left(U_{1}^{*}\left(t\right),U_{2}^{*}\left(t\right)\right)$:

(A16) $$\begin{array}{c} E\left[L_{t+1}-L_{t}+f_{t}\left(\pi_{t}\right)|Y_{t}\right]\leq \frac{1}{2}\left(V+Z\right)+\left(-\varphi_{1}+E\left[U_{1}^{*}\left(t\right)\right]\right)E\left[H_{t}\right]+\left(-\varphi_{2}+E\left[U_{2}^{*}\left(t\right)\right]\right)E\left[G_{t}\right]\\ +\frac{1}{2}\theta \left(-E\left[{Q_{t}}^{2}\right]E\left[U_{1}^{*}\left(t\right)\right]p+2E\left[Q_{t}\right]E\left[A_{t}\right]E\left[U_{2}^{*}\left(t\right)\right]\left(1-E\left[U_{1}^{*}\left(t\right)\right]p\right)+E\left[{A_{t}}^{2}\right]E\left[U_{2}^{*}\left(t\right)\right]\right)\\ +\frac{1}{2}\beta \left(-E\left[U_{2}^{*}\left(t\right)\right]E\left[{A_{t}}^{2}\right]+2E\left[A_{t}\right]E\left[K_{t}\right]\left(1-E\left[U_{2}^{*}\left(t\right)\right]\right)+E\left[{K_{t}}^{2}\right]\right)+E\left[f_{t}\left(\pi_{t}^{*}\right)\right]. \end{array}$$

Placing $E\left[K_{t}\right]=0$, $E\left[{K_{t}}^{2}\right]=\sigma^{2}$, $E\left[A_{t}\right]=0$, as well as $\varphi_{1}$ into $E\left[U_{1}^{*}\left(t\right)\right]$, and $\varphi_{2}$ into $E\left[U_{2}^{*}\left(t\right)\right]$, we get the following. It is worth noting that placing the time-average constraint on $E\left[U_{1}^{*}\left(t\right)\right]$ and $E\left[U_{2}^{*}\left(t\right)\right]$ with equality in the Lyapunov drift analysis can be justified easily by observing that the constraints must be active almost always over the *T* time horizon O(T) time instants:

(A17) $$\begin{array}{cc} E\left[L_{t+1}-L_{t}+f_{t}\left(\pi_{t}\right)|Y_{t}\right]\leq & \frac{1}{2}\left(V+Z\right)+\frac{1}{2}\theta \left(-E\left[{Q_{t}}^{2}\right]p\varphi_{1}+E\left[A_{t}\right]\varphi_{2}\right)\\ \; & +\frac{1}{2}\beta \left(-\varphi_{2}E\left[A_{t}\right]+\sigma^{2}\right)+E\left[f_{t}\left(\pi_{t}^{*}\right)\right]. \end{array}$$

Recalling that queues $A_{t}$ and $Q_{t}$ are mean rate stable, we have, $E\left[{Q_{t}}^{2}\right]=E\left[{Q_{t+1}}^{2}\right]$ and $E\left[{A_{t}}^{2}\right]=E\left[{A_{t+1}}^{2}\right]$. From (A3)–(A5), we can get the expectation of $A_{t}^{2}$ and $Q_{t}^{2}$ as

(A18) $$\begin{array}{c} E\left[{A_{t}}^{2}\right]=\frac{\sigma^{2}}{\varphi_{2}} \end{array}$$

(A19) $$\begin{array}{c} E\left[{Q_{t}}^{2}\right]=\frac{\sigma^{2}}{p\varphi_{1}}. \end{array}$$

As a result, (A17) can be simplified as follows:

(A20) $$\begin{array}{cc} E\left[L_{t+1}-L_{t}+Rf_{t}\left(\pi_{t}\right)|Y_{t}\right]\leq & \frac{1}{2}\left(V+Z\right)+\frac{1}{2}\theta \left(-\frac{\sigma^{2}}{p\varphi_{1}}p\varphi_{1}+\frac{\sigma^{2}}{\varphi_{2}}\varphi_{2}\right)\\ \; & +\frac{1}{2}\beta \left(-\varphi_{2}\frac{\sigma^{2}}{\varphi_{2}}+\sigma^{2}\right)+E\left[f_{t}\left(\pi_{t}^{*}\right)\right]\\ = & \frac{1}{2}\left(V+Z\right)+E\left[f_{t}\left(\pi_{t}^{*}\right)\right]. \end{array}$$

Now take the summation of the total T-time slot on both sides of (A20) and we have

(A21) $$\begin{array}{cc} E\left[L_{T}-L_{0}+\sum_{t=0}^{T-1}f_{t}\left(\pi_{t}\right)|Y_{t}\right]\leq & \frac{1}{2}\left(V+Z\right)T+\sum_{t=0}^{T-1}E\left[f_{t}\left(\pi_{t}^{*}\right)\right]. \end{array}$$

Note that $L_{T}\geq 0$ and $\frac{L_{0}}{T}=0$; we then divide *T* on both sides of (A21) to get the time averaged result

(A22) $$\begin{array}{cc} \frac{1}{T}E\left[\sum_{t=0}^{T-1}f_{t}\left(\pi_{t}\right)|Y_{t}\right]\leq & \frac{1}{2}\left(V+Z\right)+\frac{1}{T}\sum_{t=0}^{T-1}E\left[f_{t}\left(\pi_{t}^{*}\right)\right]. \end{array}$$

Finally, divide R on both side of (A22) to convert $f_{t}$ into target function

(A23) $$\begin{array}{c} P_{1}^{*}\left(\pi_{t}\right)\leq P_{2}\left(\pi_{t}\right)\leq \frac{V+Z}{2R}+P_{1}^{*}\left(\pi_{t}\right). \end{array}$$
